# Local destruction of superconductivity by non-magnetic impurities in mesoscopic iron-based superconductors

**DOI:** 10.1038/ncomms8614

**Published:** 2015-07-03

**Authors:** Jun Li, Min Ji, Tobias Schwarz, Xiaoxing Ke, Gustaaf Van Tendeloo, Jie Yuan, Paulo J. Pereira, Ya Huang, Gufei Zhang, Hai-Luke Feng, Ya-Hua Yuan, Takeshi Hatano, Reinhold Kleiner, Dieter Koelle, Liviu F. Chibotaru, Kazunari Yamaura, Hua-Bing Wang, Pei-Heng Wu, Eiji Takayama-Muromachi, Johan Vanacken, Victor V. Moshchalkov

**Affiliations:** 1 INPAC—Institute for Nanoscale Physics and Chemistry, KU Leuven, Celestijnenlaan 200D, Leuven B-3001, Belgium; 2 Research Institute of Superconductor Electronics, Nanjing University, Nanjing 210093, China; 3 National Institute for Materials Science, 1-1 Namiki, Tsukuba 305-0044, Japan; 4 Physikalisches Institut-Experimentalphysik II and Center for Collective Quantum Phenomena in LISA^+^, Universität Tübingen, Auf der Morgenstelle 14, Tübingen D-72076, Germany; 5 Electron Microscopy for Materials Research (EMAT), University of Antwerp, Groenenborgerlaan 171, Antwerp B-2020, Belgium; 6 Condensed Matter Physics, Institute of Physics, Chinese Academy of Sciences, Beijing 100190, China; 7 Division of Quantum and Physical Chemistry and INPAC-Institute for Nanoscale Physics and Chemistry, KU Leuven, Celestijnenlaan 200F, Leuven B-3001, Belgium; 8 Graduate School of Chemical Science and Engineering, Hokkaido University, Hokkaido 060-0810, Japan; 9 WPI-MANA, National Institute for Materials Science, 1-1 Namiki, Tsukuba 305-0044, Japan

## Abstract

The determination of the pairing symmetry is one of the most crucial issues for the iron-based superconductors, for which various scenarios are discussed controversially. Non-magnetic impurity substitution is one of the most promising approaches to address the issue, because the pair-breaking mechanism from the non-magnetic impurities should be different for various models. Previous substitution experiments demonstrated that the non-magnetic zinc can suppress the superconductivity of various iron-based superconductors. Here we demonstrate the local destruction of superconductivity by non-magnetic zinc impurities in Ba_0.5_K_0.5_Fe_2_As_2_ by exploring phase-slip phenomena in a mesoscopic structure with 119 × 102 nm^2^ cross-section. The impurities suppress superconductivity in a three-dimensional ‘Swiss cheese'-like pattern with in-plane and out-of-plane characteristic lengths slightly below ∼1.34 nm. This causes the superconducting order parameter to vary along abundant narrow channels with effective cross-section of a few square nanometres. The local destruction of superconductivity can be related to Cooper pair breaking by non-magnetic impurities.

## Abstract (web summary)

The pairing symmetry of the wavefunction in high-*T*_c_ iron-based superconductors remains not completely understood. To shed light on this problem, here the authors investigate the local destruction of superconductivity by introducing Zn impurities in the BKZn iron arsenide compound.

## Graphical abstract

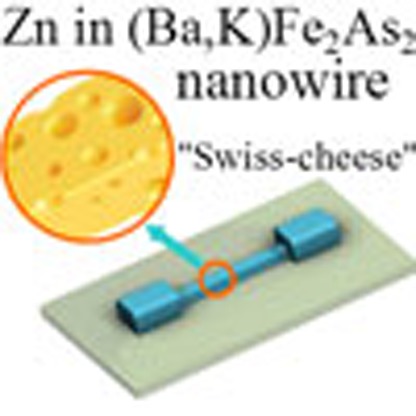

For the newly discovered high-*T*_c_ Fe-based superconductors^1^, it is essential to elucidate the pairing symmetry of the superconducting wave function^2^, for which the multi-gapped *s*-wave is generally acceptable. Possible candidates include the unconventional *s*_±_-wave with sign-reversal^3^ and the conventional *s*_++_-wave without sign-reversal^4^. According to Anderson's theorem^5^,^6^,^7^,^8^, a few at% of non-magnetic impurities can act as strong scattering centres and dramatically suppress superconductivity by pair breaking in the case of an anisotropic gap, for example, in a *d*-wave^9^ or *s*_±_ wave^2^,^3^ superconductor. Our previous experiments demonstrated that non-magnetic Zn impurities can suppress the transition temperature *T*_c_ of the 122-type Fe-based superconductors^10^, while the magnitude of the *T*_c_ suppression is lower than expected for the *s*_±_-wave scenario^4^,^11^. On the basis of these results, recent theoretical studies proposed that the suppression of superconductivity could be attributed to various effects apart from pair breaking, such as localization^12^ or disorder^13^,^14^. Furthermore, orbital fluctuations, as the possible origin of the *s*_++_ state, could be suppressed by lifting the orbital degeneracy near impurity atoms^15^, which would also lead to a reduction in *T*_c_. Therefore, the corresponding theoretical calculation of *T*_c_ suppression^10^,^11^ cannot support the pair breaking by non-magnetic impurities as the responsible mechanism directly, which requires further understanding on the role of Zn.

The impurity ions of Zn^2+^ behave as spinless centres, which may have induced moments of *s*=2 on the Fe sites (the ‘Kondo-hole problem'). Nuclear magnetic resonance (NMR) can probe nuclei coupled to the superconducting Fe_2_*X*_2_ planes to yield information on the local magnetic structure. Kitagawa *et al.*^16^ studied the Zn-substituted LaFeAsO_0.85_ polycrystal using ^75^As and ^139^La NMR and nuclear quadrupole resonance, and found that the suppression of superconductivity by Zn is not due to the change of the normal-state properties, but due to a strong non-magnetic pair-breaking effect on superconductivity. On a local-scale of suppression, the Zn ions can exclude the supercurrent of an unconventionally gapped superconductor within an area of 
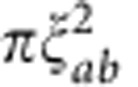
, which results in a two-dimensional (2D) ‘Swiss cheese'-like supercurrent distribution; here *ξ*_*ab*_ is the in-plane coherence length of the superconductor. This model was proposed based on the in-plane Zn-doping studies of cuprate superconductors by scanning tunnelling spectroscopy (STM)^17^ and muon spin relaxation experiments^18^,^19^, while such experiments on the iron pnictides are still in progress^20^. Since the Fe-based 122 compounds possess nearly isotropic properties^21^, the superconducting order parameter *ψ* can also fluctuate along the *c* axis. Therefore, it is essential to study both the in-plane and out-of-plane effects of Zn ions on local superconductivity, which can hardly be observed by STM or muon spin relaxation.

Local destruction of superconductivity by Zn impurities will result in a considerable suppression of the superconducting volume fraction. Thus, it is promising to study this behaviour in mesoscopic samples, in which local effects should be more pronounced^22^. For a one-dimensional (1D) superconducting system, it has been proposed that *ψ* may be spatially or temporally modulated along its length at finite temperature *T*. In this case, resistive behaviour is induced by thermally activated events, which cause *ψ* to shrink to zero and slip its phase by 2*π*. Such a process is denoted as thermally activated phase-slip^23^,^24^,^25^,^26^,^27^,^28^,^29^,^30^. Strictly speaking, it is hard to obtain a 1D superconductor experimentally with a diameter less than the characteristic length *ξ*. A quasi-1D system with corresponding dimensions being smaller than 
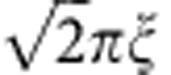
, is more feasible^25^,^26^,^27^,^28^. For low-*T*_c_ superconductors with coherence lengths *ξ*(*T*) up to a few micrometres, the 1D regime has been reached experimentally via micro- and/or nanopatterning techniques, and phase-slip processes have been observed^31^,^32^,^33^,^34^. However, this approach seems to be rather challenging for the high-*T*_c_ superconductors where *ξ* is extremely small (∼2 nm or less) and comparable to the size of just one unit cell for both cuprates and the Fe-based superconductors^1^,^10^. Surprisingly, recent measurements on a YBaCu_3_O_7−*δ*_ (YBCO) nanobridges (50 × 100 nm^2^ in cross-section) demonstrated a pronounced thermal phase-slip behaviour^35^. Since cuprate nanostructures are quite susceptible to degradation induced by chemical and thermal instability during nanopatterning, the observed phase-slip behaviour may have been dominated by inhomogeneities in the YBCO nanobridges. In contrast to this, for the Ba_0.5_K_0.5_Fe_2_As_2_ (BK) system, one can hardly observe any degradation by the patterning process^36^. This makes the system quite promising for studies of the impact of impurities on the superconducting properties in BK nanostructures.

Here we present an approach to study the effects of local destruction of superconductivity by introducing non-magnetic Zn impurities into iron arsenide Ba_0.5_K_0.5_Fe_1.94_Zn_0.06_As_2_ (BKZn) mesoscopic superconducting structures. Phase-slip behaviour was observed in nanobridges of width *W* and thickness *h* with cross-section areas *A*=*W* × *h* down to 119 × 102 nm^2^. We propose that Zn suppresses superconductivity in a three-dimensional (3D) ‘Swiss cheese'-like fashion, where *ψ* fluctuates along abundant narrow channels, which are supposed to be a few nanometres wide, close to the size of *ξ*.

## Results

### Transport properties

**Figure 1:**
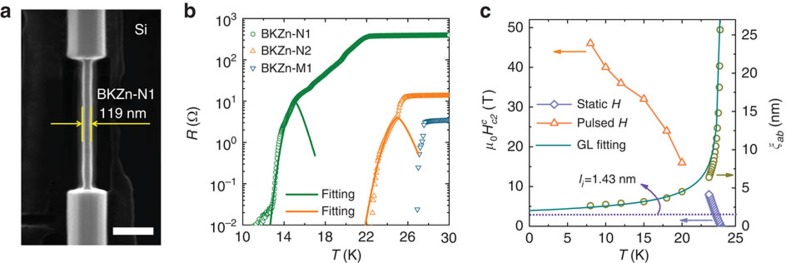
Image of the nanobridge and transport properties. (**a**) Scanning electron microscopy image of nanobridge BKZn-N1. Scale bar, 500 nm. (**b**) *R*(*T*) curves measured in zero magnetic field for nanobridges BKZn-N1, BKZn-N2 and microbridge BKZn-M1, which reveal different cross-sectional area (see, for example, Table 1). The bias current for each sample was 10 μA. Open symbols are experimental data, and solid lines are fitting results from the thermal activated phase-slip model proposed by Little^23^. (**c**) 
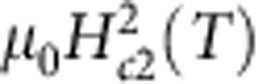
 and *ξ*_*ab*_(*T*) measured on microbridge BKZn-M1. 
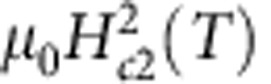
 is extracted from the resistive transition points at 90% of the normal state resistivity *ρ*_*n*_, as determined from a Physical Properties Measurement System in static fields (<9 T, see Supplementary Fig. 3) and from a pulsed field set-up up to 52 T (Supplementary Fig. 5). *ξ*_*ab*_(*T*) is estimated from the Ginzburg–Landau formula for an anisotropic 3D superconductor 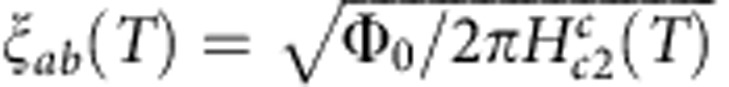 (ref. 21); Φ_0_ is the magnetic flux quantum. We also estimated *ξ*_*ab*_(*T*) by the Ginzburg–Landau relation 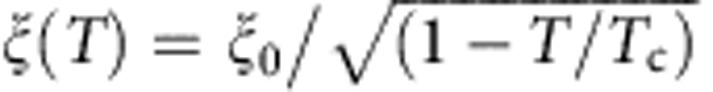 as shown by the dark cyan line. The horizontal violet dotted line shows the mean distance *l*_*i*_ between neighbouring Zn ions.

Figure 1a shows a scanning electron microscopy image of the nanobridge BKZn-N1 (width *W*=119 nm, thickness *h*=102 nm). The resistance *R* versus *T* curves of nanobridges BKZn-N1 and BKZn-N2 (*W*=290 nm, *h*=315 nm), and of the microbridge BKZn-M1 (*W*=2000, nm, *h*=373 nm) are given in Fig. 1b. The microbridge BKZn-M1 exhibits a sharp superconducting transition, indicating bulk behaviour. On the other hand, the *R*(*T*) curves of the nanobridges show several steps, which are particularly pronounced for BKZn-N1 with the smallest cross-section *A*. The transition width Δ*T*_c_∼11 K of BKZn-N1 is also larger than that of the other bridges (for example, Δ*T*_c_∼3 K for BKZn-N2). Similar steps and a broadened Δ*T*_c_ in the *R*(*T*) curves were frequently observed in low-*T*_c_ nanowires 31,32,33,34 and high-*T*_c_ YBCO nanobridges^35^, and were explained by thermally activated phase-slips.

**Figure 2:**
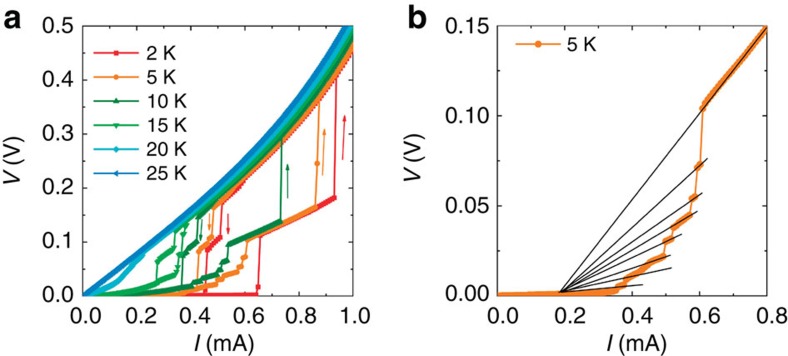
current-voltage characteristics (IVCs) of nanobridge BKZn-N1. (**a**) current-voltage characteristics (IVCs) of nanobridge BKZn-N1 measured at different *T* in zero magnetic field. Arrows indicate bias current sweep directions. (**b**) Enlarged view for sweep-up IVC at 5 K. Once extrapolating each successive branch linearly, all branches intersect at *V*=0, representing a phase-slip centre as described by Tinkham^24^.

Figure 2a shows the current–voltage characteristics (IVCs) of the Zn-doped nanobridge BKZn-N1 taken at *T*=2–25 K. On increasing the bias current from zero, the sample switches to an intermediate resistive state (for example, at a critical current *I*_c_=0.64 mA at *T*=2 K). By further increasing *I*, a second jump to the normal state appears. When the current is swept down, the sample retraps from the normal to the intermediate resistive regime at a retrapping current *I*_r_ and finally to the superconducting state after one or two intermediate states. The steps in the IVCs and the *R*(*T*) curves exhibit characteristics typical for phase-slips. The pronounced hysteresis on the IVCs can be attributed to Joule heating. The instantaneous dissipation affects the local temperature of the mesoscopic system and results in an increased probability for thermal activation^27^,^37^. According to Tinkham's theory^24^, the phase-slips can move in a homogeneous wire system (like vortices in a clean crystal), and the phase-slip occurs at a relatively higher current due to the larger critical current, where more heat will be generated to switch the wire into the normal state immediately after the occurrence of a single phase-slip. For inhomogeneous wires, however, a phase-slip can occur and it can be pinned at a weak link. Such a phase-slip would be acting at a lower current and thus can exist without overheating the whole wire. For the undoped BK nanobridges, however, IVCs demonstrated switching from the superconducting to the voltage state with absence of any intermediate state (Supplementary Fig. 2).

Figure 2b shows an enlarged view of the sweep-up IVC at 5 K. Once extrapolating each successive branch linearly, all branches intersect at *V*=0, representing a typical phase-slip behaviour as described by Tinkham^24^. Each step in the IVC indicates the appearance (somewhere along the nanobridge) of an additional similar localized resistance centre, where the time-average pair chemical potential 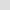 suffers a discontinuous step-like increment, 
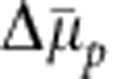
. Such a centre is usually considered as a phase-slip centre. We also simulated a IVC by applying time-dependent Ginzburg–Landau theory on a 1D system (see Supplementary Note 2 and Supplementary Fig. 9). Comparing the theoretical study with the experimental curves, we conclude that the hysteretic and stair-like behaviour presented in the experimental IVC curves are watermark indications for phase-slip centres.

**Figure 3:**
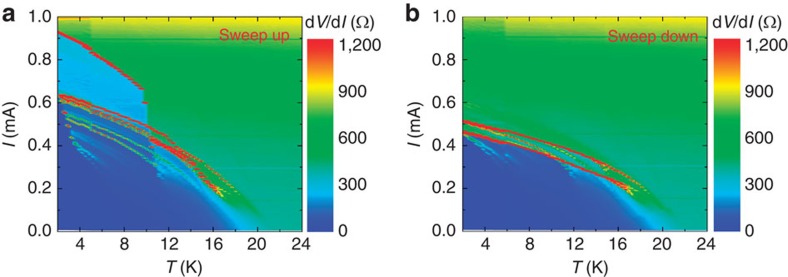
Differential resistance of the nanobridge BKZn-N1. Bias current *I* and temperature *T*-dependent differential resistance d*V*/d*I* at zero magnetic field. The data were collected from (**a**) sweep-up and (**b**) sweep-down branches. Red points indicate the critical currents at which large voltage jumps appear.

In Fig. 3, we show the differential resistance d*V*/d*I*(*I*, *T*) for the nanobridge BKZn-N1; Fig. 3a shows data taken by sweeping up the bias current and Fig. 3b shows data for the sweep-down branches of the IVCs. The red points indicate the largest values for d*V*/d*I*, corresponding to the switching currents. For the sweep-up data, we detect at *T*=2 K two intermediate resistive states, which correspond to two avalanche processes of phase-slip. For *T*>2 K, more intermediate resistive states are observed, indicating several phase-slip entrances. For the sweep-down branches, we find two major switching steps, as indicated by the two distinct red lines in Fig. 3b.

### Coherence length

Considering the general condition for the appearance of phase-slips, the cross-sectional area should be compared with 
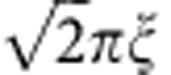
. *ξ*(*T*) can be evaluated from the upper critical field *H*_*c*2_ as 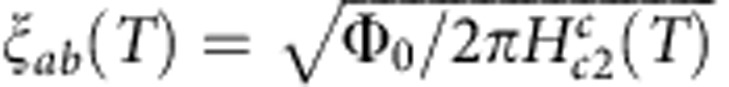 and 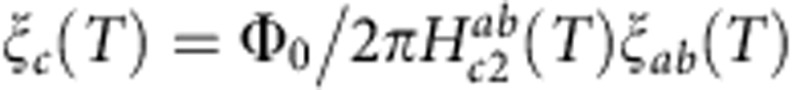 (ref. 21). We measured 
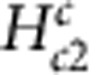
 by using static fields up to *μ*_0_*H*=8 T and pulsed fields up to 52 T, applied in the *c* direction; the results are shown in Fig. 1c together with the corresponding *ξ*_*ab*_. We also extrapolated *ξ* to *T*=0 by using the Ginzburg–Landau expression 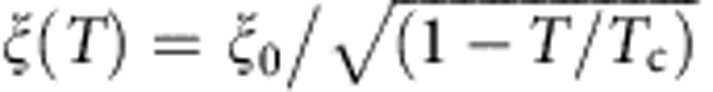. This yields *ξ*_*ab*_(0)=2.05 nm and *ξ*_*c*_(0)=1.20 nm, where the latter was determined from d.c. field measurements (Supplementary Figs 3 and 4). The values for *ξ*_*ab*_(0) and *ξ*_*c*_(0) are two orders of magnitude smaller than either the width or the thickness of our nanobridges; this suggests that the dimensions of the nanobridges are too large to allow for the generation of any phase-slips. However, since Zn induces a local destruction of superconductivity, the effective superconducting regions are likely to be shrunk into narrow channels having a relatively small effective cross-section A* within the nanobridges.

To access A*, we fit the *R*(*T*) curves by using the thermally activated phase-slip theory proposed by Little^23^. We treat the nanobridge as a quasi-1D system, where a phase-slip passes over a free-energy barrier Δ*F* proportional to the cross-sectional area, 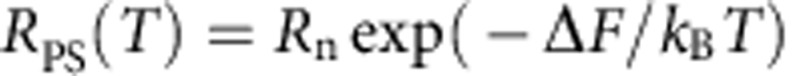 (refs 31, 32, 33, 34). Here *R*_n_ is the normal resistance, and Δ*F* is given by 
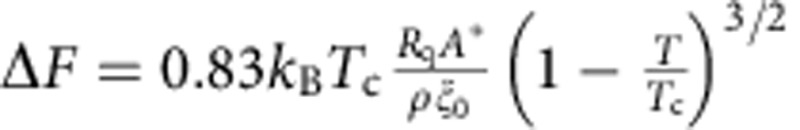
, where, *R*_q_=6.45 kΩ is the superconducting quantum resistance and *ρ*_0_ is the resistivity (we take the residual resistivity *ρ*_0_=49.98 μΩ cm from results of BKZn-M1). The fitting of *R*_PS_(*T*) to the *R*(*T*) curves is shown in Fig. 1b. From these fits, we obtain A*=8.26 and 455.27 nm^2^ for BKZn-N1 and BKZn-N2, respectively. For BKZn-N1, the obtained A* is three orders of magnitude smaller than the geometric cross-section *A* of the nanobridge, while it is less than the value of 
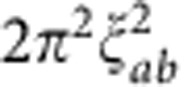
. For BKZn-N2, A* is relatively large but still two orders of magnitude smaller than its cross-section *A*. We note that the phase-slip phenomenon can only appear within a narrow temperature interval close to *T*_c_, where *ξ* is relatively large, as shown in Fig. 1c.

### Superconducting order parameter fluctuation

We can conclude that Zn plays a significant role in reducing the cross-section of superconducting channels and for the observation of the phase-slip phenomenon, that is, Zn suppresses superconductivity locally. Considering fluctuations of the superconducting order parameter *ψ*, the influence of Zn on the 1D or quasi-1D high-*T*_c_ unconventional superconductors can induce two different types of *ψ* variations, in-plane and out-of-plane.

**Figure 4:**
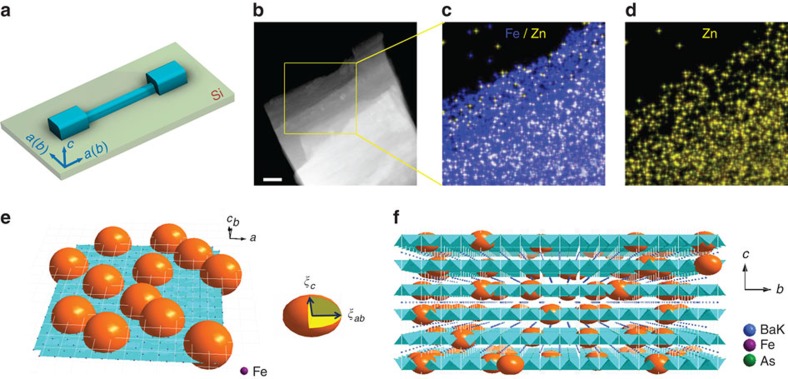
Swiss cheese model. (**a**) Transport measurement scheme along a nanobridge. The current flows along the *ab* plane. (**b**) HAADF-STEM image of a BKZn flake, where the crystal was detected along the *c* axis. Scale bar, 50 nm. (**c**,**d**) STEM-XEDS mapping for Zn/Fe and Zn distributions, respectively, within the area indicated in **b**. (**e**,**f**) Schematic representation of the 2D and 3D ‘Swiss cheese' models, respectively. The yellow oblate spheroid corresponds to the non-superconducting regions centred on Zn ions with an equatorial length *ξ*_*ab*_ and a polar length *ξ*_*c*_.

First of all, since the 122-type superconductors possess an anisotropic-layered structure, the Cooper pairs prefer to reside within the Fe_2_As_2_ superconducting planes. Previous X-ray and in-plane resistivity analysis indicated that the Zn ions were substituted on the Fe sites of Ba_0.5_K_0.5_Fe_2−*x*_Zn_*x*_As_2_ (ref. 10). Thus, the Cooper pairs can be broken and the supercurrent will be excluded from a Zn-centred area of 
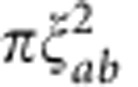
 within the planes, vitalizing the so-called 2D ‘Swiss cheese' model^18^,^19^ as shown in Fig. 4e. To explore the distribution of Zn ions within the *ab* plane of BKZn crystals, we performed high-angle annular dark-field scanning transmission electron microscopy (HAADF-STEM), as shown in Fig. 4b, together with STEM-energy-dispersive X-ray spectroscopy (STEM-XEDS) mapping as shown in Fig. 4c,d. The Zn ions were found to be homogeneously distributed within the *ab* plane of the crystal, without any indication for phase separation. Since only 3 at% of Fe ions were substituted by Zn in our crystals, we presume the idealized situation that the Zn ions are homogenously distributed within the Fe_2_As_2_ layers. Thus, a mean distance *l*_*i*_ between Zn ions can be estimated as ∼1.60 nm using 
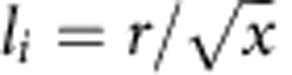
, where *x* is the Zn-doping level, and *r*=2.77 Å is the distance between the neighbouring Fe ions. *l*_*i*_ is significantly smaller than *ξ*_*ab*_(*T*), especially, near *T*_c_ (see Fig. 1c). As a result, it seems very likely, that the non-superconducting regions induced by Zn impurities inhibit superconductivity almost within the whole crystal. In addition, we estimate the in-plane non-superconducting volume fraction 
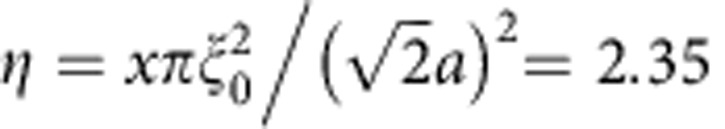
, where *a* is the lattice constant. Thus, it seems that superconductivity can hardly survive in crystals with such doping level of Zn. However, previous experiments indicated that superconductivity of BK can be strongly resistant against Zn impurities up to *x*=10 at% (ref. 36).

To investigate the local destruction area of Zn, STM experiments will be the most promising way. But, unfortunately, the STM study on the Zn-doping effect on Fe-based superconductors is still under work. The main challenge for the STM experiments is due to the technical difficulty in high-quality single-crystal fabrication and *in situ* cleaving technique. Zhu *et al.*^38^ have calculated a single non-magnetic impurity behaviour in the (K,Tl)Fe_*x*_Se_2_ superconductors for various models. The impurity-induced resonance state was found to exist only for the 
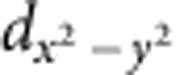
-wave pairing state, but not for the others like *s*_±_. Besides, the bound-state peak in the local density of states occurs at a non-zero energy for Fe-based systems even in the unitary limit, indicating an opposite situation from the cuprate systems. On the basis of the experimental results on Zn-doping, Chen *et al.*^39^ suggested that the in-plane *ξ* for the case of a *s*_±_ paring symmetry should be as short as two lattice sites, namely, *ξ*_Δ_∼0.55 nm. Such a short screening length was attributed mainly to the strong local Coulomb repulsion *U*, which acts on the charges. We note that the corresponding *η* is quite low, 0.17 for *x*=3 at% and *ξ*_Δ_∼0.55 nm. In this case, the *ψ* fluctuations could be strong enough to induce the motion of phase-slips, thus inhibiting the generation of thermally activated phase-slips^24^. Therefore, we argue that the characteristic length should be slightly <1.34 nm, which is estimated from the boundary condition *η*=1.

Considering the out-of-plane variations of *ψ*, since *ξ*_*c*_ perpendicular to the plane (*ξ*_*c*_(0)=1.20 nm) of the crystal is larger than the distance between each superconducting plane (*t*=0.665 nm (ref. 10)), the amplitude of *ψ* can be easily strong enough for Josephson coupling between the layers^40^, resulting in a weakly anisotropic behaviour as discussed before^21^,^36^. *ξ*_*c*_(0) of Zn-doped samples is larger than *t*=0.666 nm as well^10^, and particularly, *ξ*_*c*_(*T*) is increasing with increasing *T*. However, with Zn doping, the non-superconducting islands around the Zn ions can break the superconducting structure symmetry along the *c* axis, leading to an inhomogeneous 3D structure, for which we propose a 3D ‘Swiss cheese' model as shown in Fig. 4f, namely, stacks of 2D ‘Swiss cheese' separated by Ba/K barrier layers. The presence of non-superconducting islands acts as additional barriers enhancing the distance between the adjacent superconducting layers and weakening the Josephson coupling along the *c* axis. Consequently, we may assume that *ψ* develops along abundant narrow superconducting channels within the nanobridges, whose A* should be dramatically smaller than the cross-sectional area.

## Discussion

On the basis of the 3D ‘Swiss cheese' model, the phase-slip phenomenon could be observed in a bulk crystal once the Zn impurity is homogenously distributed within the crystal. However, we can hardly perform transport measurements on a bulk crystal because of the dramatically higher value of *I*_c_. Instead, we studied microbridges with 2 μm width and thicknesses ranging from 49.2 to 479.5 nm. Phase-slip was found for microbridges with thickness up to 415.5 nm within a narrow temperature region just below *T*_c_ (see Supplementary Fig. 6 and Supplementary Note 1). For thinner bridges, however, phase-slip phenomenon was enhanced quite substantially. Indeed, one can hardly synthesize an ideal crystal with Zn ions distributed within the Fe_2_As_2_ plane homogenously. The sheet resistance (*R*_□_) of the Zn-doped crystal is *R*_□_=*ρ*_*n*_/*t*≈211.7 Ω, which is slightly larger than that of an impurity-free crystal (*R*_□_=88.9 Ω), indicating the existence of weak localization. In addition, the existence of weak Zn clusters may induce relatively wide superconducting channels and restrict phase-slipping, which will be much more pronounced in bulk crystals and relatively wide microbridges, as compared with mesoscopic system.

The impurity-free nanobridges demonstrated thermal stability due to smaller number of defects. With introducing Zn, the nanobridges revealed several phase-slips. We propose that Zn suppresses superconductivity in a 3D ‘Swiss cheese'-like pattern, where the order parameter is restricted to be developed along abundant narrow superconducting channels. Considering that the order parameter has zero value in the point of each impurity and since the magnitude of the order parameter can only change within its characteristic length scale, we can exclude the spherical regions with radius *ξ* around each impurity from the superconducting condensate, for which the detail discussion on order parameter along the nanobridge is introduced in Supplementary Fig. 8 and Supplementary Note 2. We estimated the effective cross-section of the superconducting channels as a few nanometres according to the mechanism of thermally activated phase-slips. This cross-section value is consistent with the magnitude of the temperature-dependent coherence length supporting the proposed model.

For a conventional superconducting gap like *s*_++_, the non-magnetic impurity ions work as point defects, but do not affect the Cooper pairs. Oppositely, the Zn ions can induce local destruction of superconductivity for the unconventional *s*_±_ pairing symmetry, and consequently result in phase-slip phenomenon in the BKZn nanobridges or even microbridges. The local destruction may provide an evidence for the pair-breaking effect of non-magnetic impurities and the unconventional *s*_±_ pairing symmetry for the iron pnictide superconductors. Meanwhile, the observation of an unusual large A*, especially for BKZn-N2, which could be due to the induced competing order around Zn impurities, suggests that further experiments might be necessary to achieve a better understanding of the nature of the superconducting gap symmetry in iron-pnictides superconductors, as well as the role of Zn impurity.

## Methods

### Crystal growth

The BK (Ba_0.5_K_0.5_Fe_2_As_2_) and BKZn (Ba_0.5_K_0.5_Fe_1.94_Zn_0.06_As_2_) single crystals were grown using high-pressure technique as described elsewhere^36^: the stoichiometric mixture of BaAs, KAs, FeAs, Fe and Zn was placed in a tantalum capsule with an *h*-BN inner, and compressed at 3 GPa in a belt-type high-pressure apparatus and heated at 1,300 °C for 4 h. The elemental concentration of BKZn was confirmed by energy dispersive X-ray spectroscopy (Supplementary Fig. 1).

### Nanobridge fabrications

The single crystals were cleaved along their *c* axis into flakes with thickness down to a few hundred nanometres, and then were glued onto Si substrates, with their *ab* plane parallel to the substrate surface, by using a thin layer of epoxy. The crystals were then fabricated as microbridges as following process^10^: (i) Au depositing onto the crystal; (ii) annealing at 200 °C for 24 h under nitrogen atmosphere; (iii) photolithography patterning on the crystal; (iv) argon ion milling the sample into a microbridge; (v) removing the photoresist by acetone and connecting the electrodes with silver paste; and (vi) argon ion milling the whole sample to remove the Au layer. The thin crystals were patterned into microbridges with width *W*=2 μm, length *L*=10 μm and thickness *h*=100–400 nm. Subsequently, some of the microbridges were cut by a focused ion beam system equipped with a Ga ion source (FEI Dual beam Strata 235) to produce constrictions within the bridges with *W* down to 119 nm and different *L*. The focused ion beam milling was based on a procedure used earlier for YBCO thin films^41^. Here we present data on micro- and nanobridges with dimensions given in Table 1, where the nanobridges were patterned by ion milling with a focused Ga beam, while not for the microbridges. Figure 1a shows a scanning electron microscopy image of the nanobridge BKZn-N1. The thickness was confirmed from the resistance measurement of the microbridge^10^.

## Additional information

**How to cite this article:** Li, J. *et al.* Local destruction of superconductivity by non-magnetic impurities in mesoscopic iron-based superconductors. *Nat. Commun.* 6:7614 doi: 10.1038/ncomms8614 (2015).

## Supplementary Material

Supplementary Information Supplementary Figures 1-9, Supplementary Notes 1-2 and Supplementary References

## Figures and Tables

**Table 1:** Dimensions of all micro- and nanobridges.

**Samples**	**Materials**	***W*** **(nm)**	***L*** **(nm)**	***h*** **(nm)**
BK-N1	Ba_0.5_K_0.5_Fe_2_As_2_	340	402	105
BKZn-N1	Ba_0.5_K_0.5_Fe_1.94_Zn_0.06_As_2_	119	1,452	102
BKZn-N2	Ba_0.5_K_0.5_Fe_1.94_Zn_0.06_As_2_	290	1,558	315
BKZn-M1	Ba_0.5_K_0.5_Fe_1.94_Zn_0.06_As_2_	2,000	10,000	373
