# Supplementary material for: Local destruction of superconductivity by non-magnetic impurities in mesoscopic iron-based superconductors
Source: Nat Commun. 2015 Jul 3;6:7614. doi: 10.1038/ncomms8614 (PMC4506518; doi:10.1038/ncomms8614)
Supplement: Supplementary Information — Supplementary Figures 1-9, Supplementary Notes 1-2 and Supplementary References [file ncomms8614-s1.pdf]

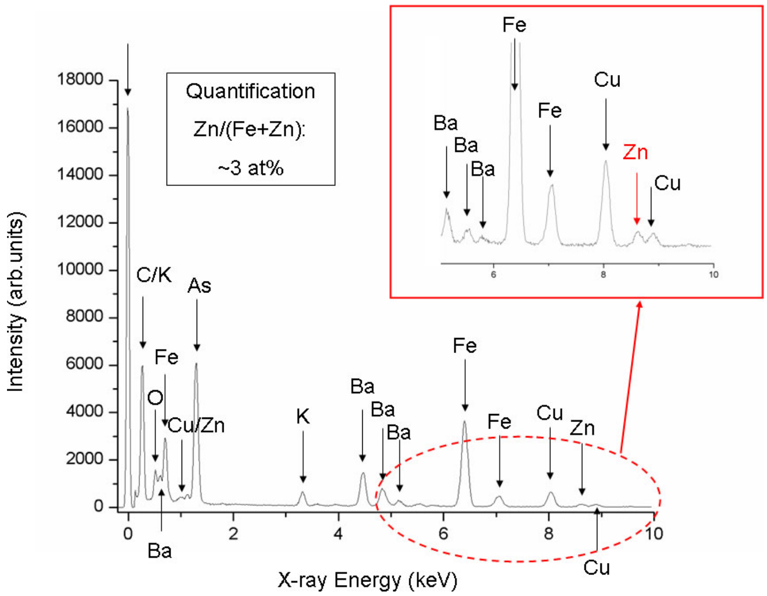

Supplementary Figure 1. STEM-EDX analysis for Zn distribution in an indicated area shown in Figure 4b. For the STEM-EDX characterization experiments, the BKZn crystals were firstly cleaved into flakes with thicknesses from 100 to 400 nm, and the as-prepared BKZn flakes were crushed and dispersed in ethanol before dropped onto a carbon film supported TEM grid. High angle annular dark field scanning transmission electron microscopy (HAADF-STEM) and energy dispersive X-ray spectroscopy (EDX) were performed using a FEI Titan "cubed" microscope, equipped with an aberration corrector for the probe-forming lens and a Super-X EDX detector, operated at 200 kV. The convergence semi-angle was approximately 21.3 mrad.

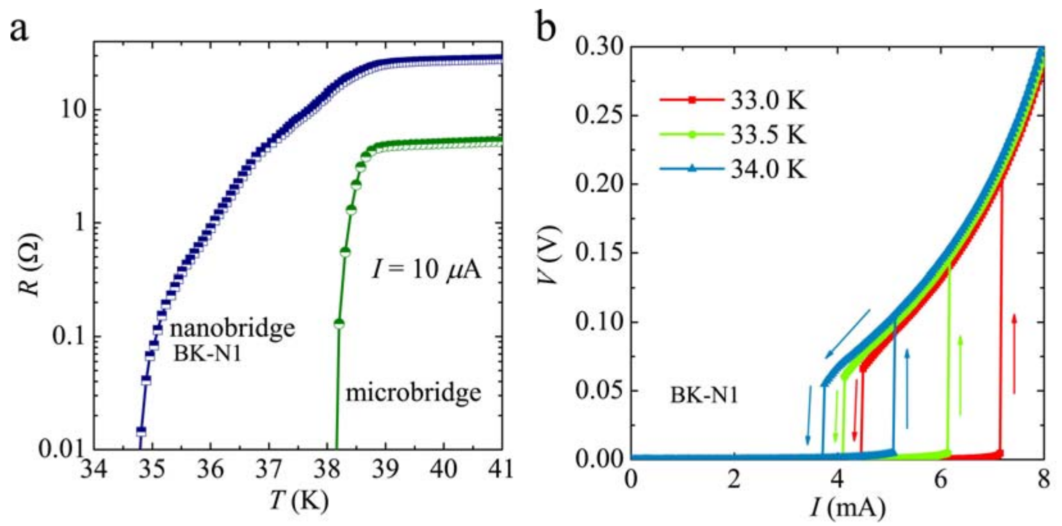

Supplementary Figure 2. (a) Resistance R vs temperature T for the undoped $Ba_{0.5}K_{0.5}Fe_2As_2$ microbridge and nanobridge BK-N1 (W = 340 nm, h = 105 nm). (b) Current-voltage characteristics (IVCs) of the nanobridge BK-N1, measured at variable T in zero magnetic field. The shape of the IVCs in the voltage state is typical for a flux-flow process, and switching from the superconducting to the voltage state yields critical current densities J_c=20.06, 17.19, and 14.23 MA/cm^2, for T = 33.0, 33.5 and 34.0 K, respectively. These values are in accordance with the depairing current density of BK microbridges [1]. The IVCs are monotonic with absence of any intermediate state.

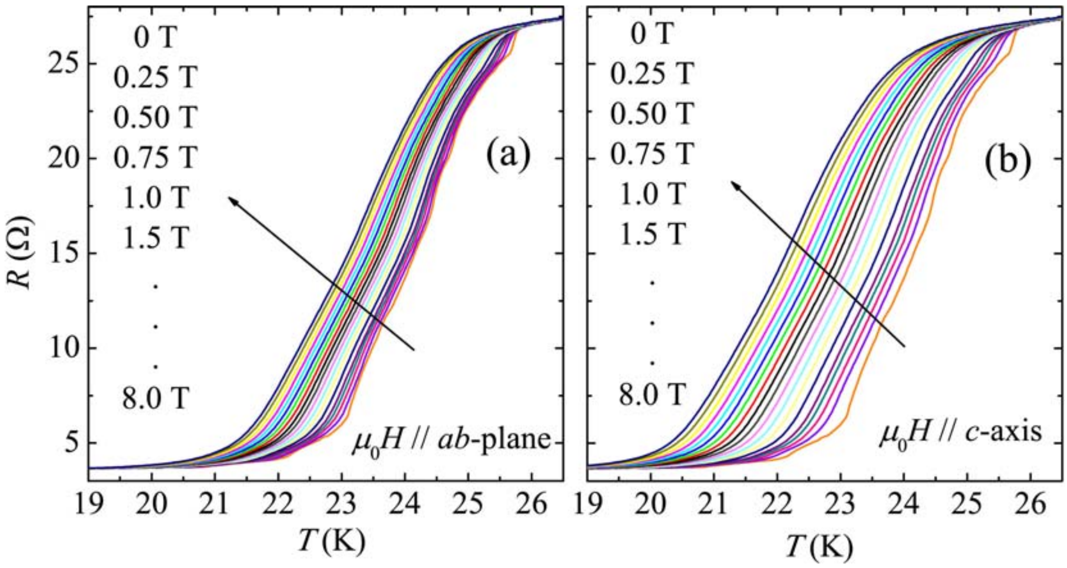

Supplementary Figure 3. Temperature-dependent magnetoresistance R for the nanobridge BKZn-N3 with cross-section of 165×244 nm^2. The magnetic field is ranging from 0 to 8 T and applied in the ab-plane for (a) and along the c-axis for (b). The bias currents were 10 μA for all measurements. The $R_{ab}(T, H)$ shows slightly anisotropic T_c suppression.

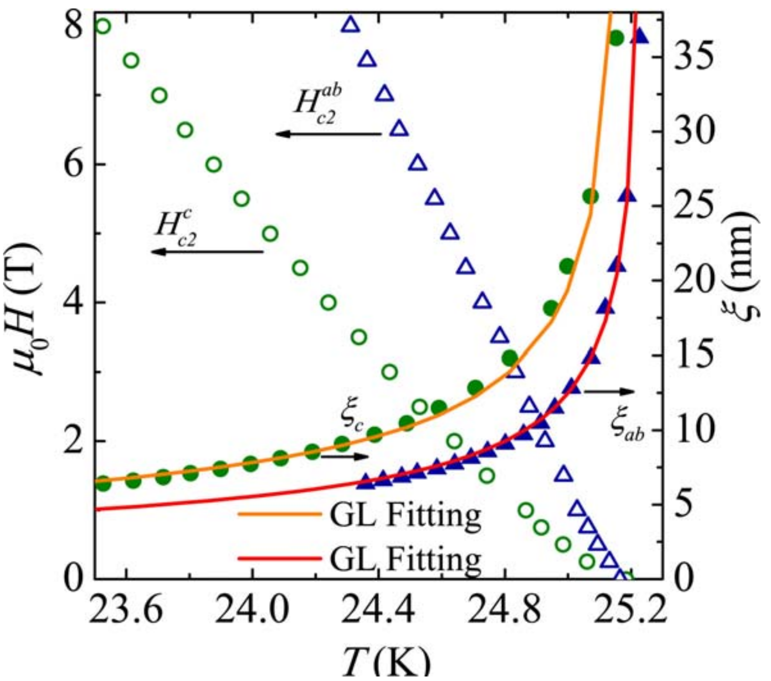

Supplementary Figure 4. Temperature-dependent H_{c2} and ξ for nanobridge BKZn-N3. Here the H_{c2} is defined from the resistivity transition points at 90% of ρ_n(29 K) in Supplementary Figure 3, and ξ_{ab} is estimated by the Ginzburg-Landau formula for an anisotropic three-dimensional superconductor $\xi_{ab}(T)=\sqrt{\Phi_0/2\pi H_{c2}^{c}(T)}$. We also fit $\xi_c(T)$ by Ginzburg-Landau (GL) theory, $\xi_c(T)=\Phi_0/2\pi H_{c2}^{ab}(T)\xi_{ab}(T)$ as shown by the orange line. Hence, we can roughly estimate ξ_{ab} (0 K) = 2.15 nm and ξ_c (0) = 1.20 nm.

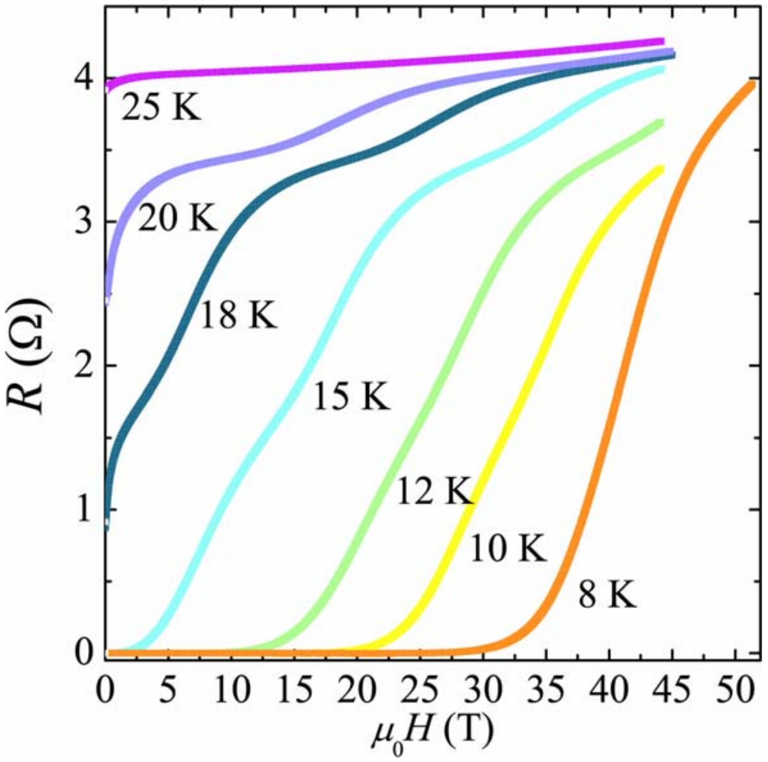

Supplementary Figure 5. Temperature-dependent magnetoresistance for a $Ba_{0.5}K_{0.5}Fe_{1.94}Zn_{0.06}As_2$ microbridge with thickness of 221 nm. The magnetic pulsed field is up to 52 T, and applied along the *c*-axis. The bias currents were 100 μA for all measurements.

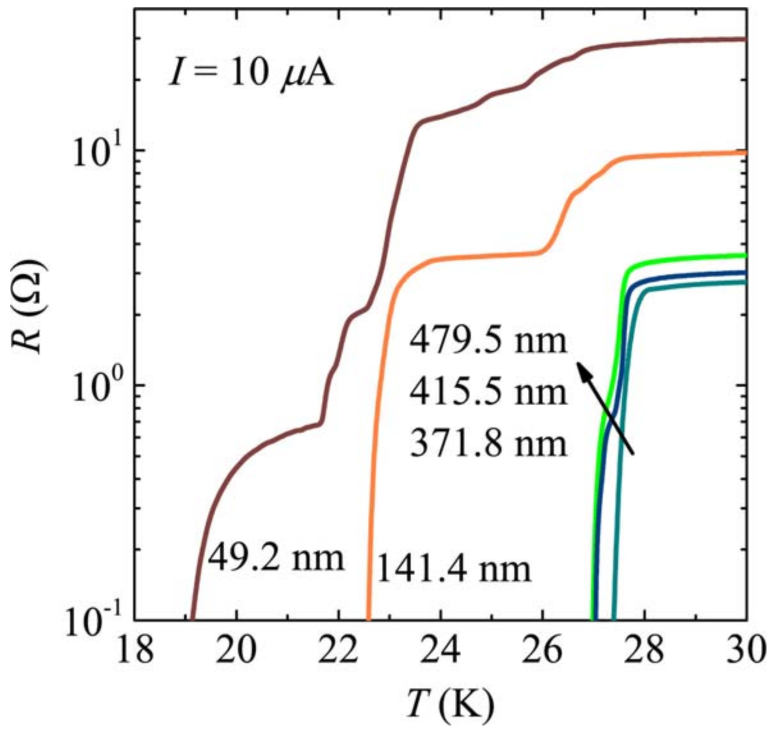

Supplementary Figure 6. Temperature dependence of R for $Ba_{0.5}K_{0.5}Fe_{1.94}Zn_{0.06}As_2$ microbridges with thicknesses h of 49.2, 141.4, 371.8, 415.5, and 479.5 nm. For the relatively large h of 479.5 nm, the T_c-onset is 28 K and the transition width ΔT_c is ~0.5 K, suggesting a bulk-like property. For h = 371.8 and 415.5 nm, the $R(T)$ curves demonstrate a weak step after the sharp drop from T_c -onset. When h is reduced to 141.4 nm, a pronounced plateau-like step is observed at 90% of the normal state resistance R_n, and the width of the plateau is as long as 3.5 K. Further reducing h till 49.2 nm, one can obtain about seven steps.

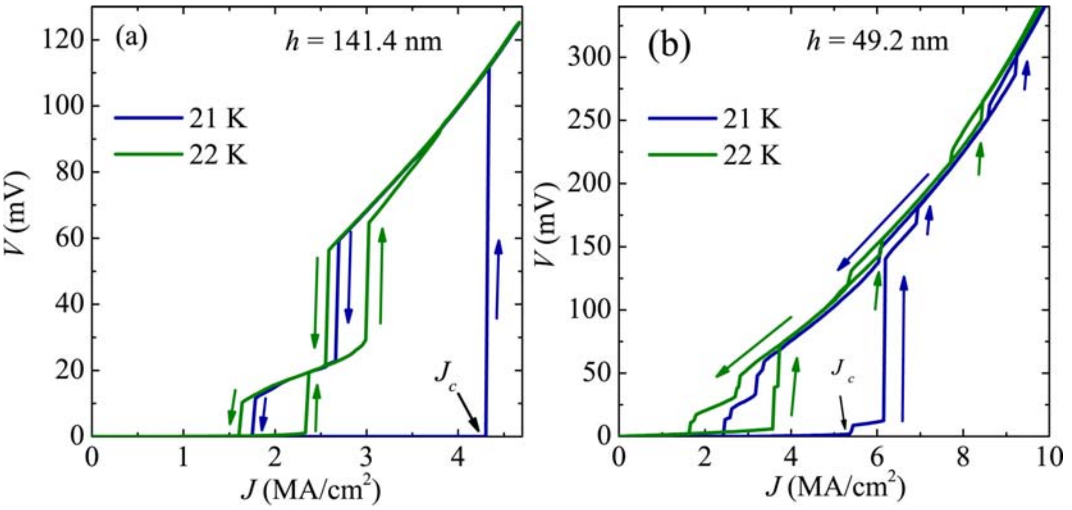

Supplementary Figure 7. Current density dependent voltage characteristics (JVCs) for the microbridges with width of 2 μm, and h of 141.4 nm for (a) and 49.2 nm for (b). The colored arrows indicate current bias increasing and decreasing process.

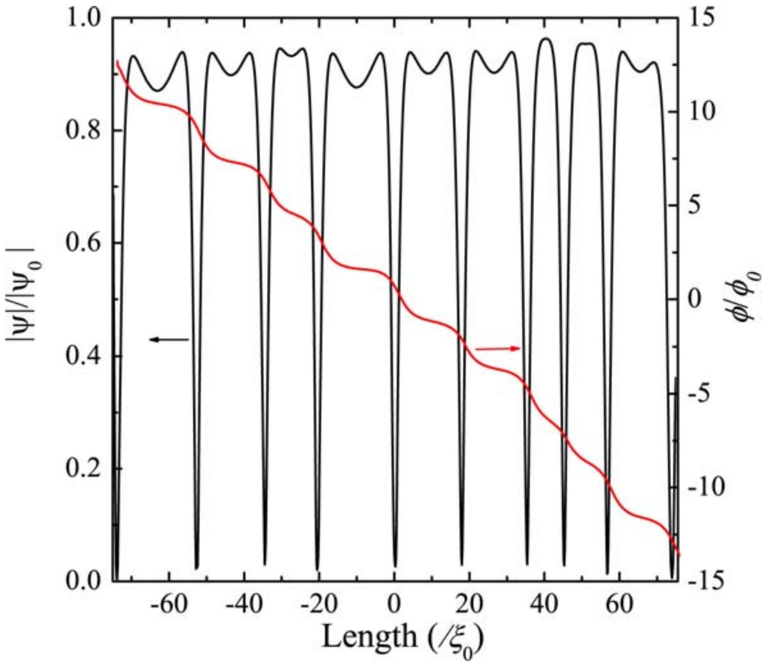

Supplementary Figure 8. Order parameter distribution, with axes on the left, and electrical potential, with axes on the right, along the one dimensional domain, corresponding to $j = 0.4j_0$.

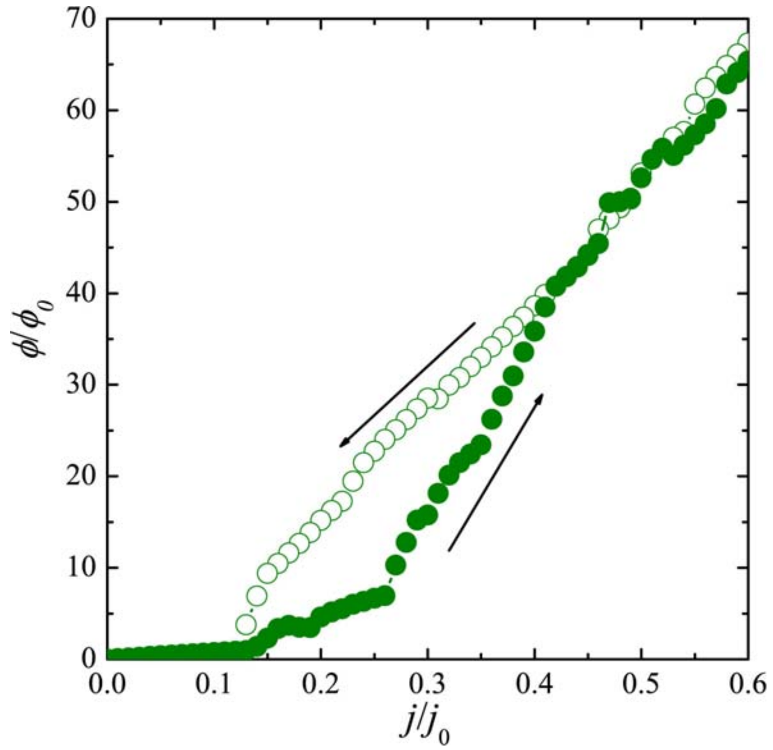

Supplementary Figure 9. Electrical potential as function of the applied current for a one dimensional superconductor with length, $L = 150\xi_0$. Right to left (left to right) arrows indicate that the *I-V* curve below the arrows was taken starting at a high (low) value of current, slightly above critical current (much below critical current).

Supplementary Note 1: phase-slips in superconducting BKZn microbridges

At 21 K, the voltage state of the 141.4 nm thick microbridge (see **Supplementary Figure 7(a)**) is switched from the superconducting to the normal state directly with absence of flux-flow process, and the critical current density J_c shows a high value of 4.3 MA/cm^2, which is close to the Ginzburg-Landau depairing current density J_{dp}^{GL} [1]. When the current is swept down, the voltage retraps from the normal to superconducting states, followed by a hysteretic resistance state. After slightly increasing T to 22 K, current sweep-up leads to voltage jumps from superconducting to the hysteretic resistance state with a slight flux-flow [4], and then to the normal state. The returning branch locates on the hysteretic resistance state as well. For the thinner microbridges with h = 49.2 nm, successive steps are observed for both current sweep-up and sweep-down processes as shown in Supplementary Figure 7(b). The single and multiple steps on the JVCs are consistent with that of the $R(T)$ curves for each microbridge, indicating the presence of phase-slip center(s) or line(s). On the other hand, the pronounced hysteresis on JVCs exhibits the kinetics of the Joule heat liberated or absorbed processes, which dominate the instantaneous dissipations of local temperature of the mesoscopic system and result in heat-flow. Consequently, they can feed back to influence the

phase-slip kinetics (see Tinkham *et al.* [5] and Pekker *et al.* [6]).

Supplementary Note 2: simulations on the phase-slips in superconducting nanowires

A nanowire with the width (W) and height (h) of the order of $\xi(T)$ and length $L >> \xi$, can be consider as nearly one-dimensional object. If we apply to it a current slightly below critical current we may obtain a phase slippage that can be observed as a jump in the *I-V* curves [7]. This is called a phase slip center since it is located on a point of the nearly one-dimensional nanowire [7]. In thicker nanowires, where $W >> \xi(T)$, phase slips were also observed when $W << \lambda_{eff}$ [8] which present similar *I-V* curves to the ones of phase slip centers. These are phase slips lines along which the order parameter is zero. One of the most noticeable features of phase slips, when compared to other dissipation mechanisms, is the hysterical behavior in the *I-V* curves. For example, changes in conductivity due to fluctuation mechanisms, e.g. Aslamazov-Larkin and Maki-Thompson, do not present hysteric behavior in *I-V* curves since they are statistical corrections to the average value of conductivity that depend only on thermodynamical conditions [9], like temperature.

In our samples, like BKZn-N1, the length is of the order of hundreds of ξ and the width and height are of the order of tenths of ξ. We are far from the thin nanowire regime, although $\lambda_{eff}(T) >> W$, in the BKZn-N1 case $2\lambda^2(0)/h = 635$ nm. As discussed before in this article and as proposed in Ref. [9] and corroborated with experimental evidences, the charge carriers around Zn impurities do not participate in the superconducting condensate. Considering that the order parameter has zero value in the point of each impurity and since the magnitude of the order parameter can only change within its characteristic length scale, we can exclude the spherical regions with radius ξ around each impurity from the superconducting condensate. This creates a geometry/topology that resembles a "Swiss cheese" which gave the name to this model. In case of an applied current, the superconducting electrical charges are transported along 1D percolation channels shaped by the impurities in the samples.

To study phase slips, we considering a similar model to the one presented in Ref. [10], that we will further describe. We solved the dimensionless generalized time dependent Ginzburg-Landau [11],

$$\frac{u}{\sqrt{1+\gamma^2|\Psi|^2}}\left(\frac{\partial}{\partial t}+i\phi+\frac{\gamma^2}{2}\frac{\partial|\Psi|^2}{\partial t}\right)\Psi=\left(\vec{\nabla}-i\vec{A}\right)^2\Psi+\left(1-T-|\Psi|^2\right)\Psi,$$

in a one-dimensional line of length L. In this equation Ψ is the order parameter, φ is the electrical potential, $\vec{A}$ is the magnetic field, i is the imaginary constant, and γ and u are two phenomenological parameters related with the electron-phonon relaxation time and the cleanness/dirtiness of the sample, respectively. The generalized time-dependent Ginzburg-Landau equation is complemented by the equation for the electric field:

$$\Delta\phi=\vec{\nabla}\cdot\mathrm{Im}\left[\Psi*\left(\vec{\nabla}-i\vec{A}\right)\Psi\right].$$

In these equations the length is given in units of $\xi(0)=\sqrt{8k_B T_c/\pi\hbar D}$, where, T in units of T_c, Ψ in units of $\Psi(0)=4k_B T_c/\sqrt{u\pi}$, (bulk value at zero temperature), φ in units of $\phi_0=\hbar/\left(2e\tau_{GL}\left(0\right)\right)$, $\vec{A}$ in units of $\phi_0/\left(2\pi\xi\left(0\right)\right)$, the current j is in units of $j_0=\sigma_n\hbar/2e\tau_{GL}(0)\xi(0)$, where k_B, T_c, $\hbar$, $\tau_{GL}(0)$, σ_n are the Boltzmann constant, the critical temperature of the superconductor condensate, the Planck constant, the diffusion constant, the Ginzburg-Landau relaxation time at zero temperature and the normal state conductivity, respectively. Since are considering a one-dimensional geometry and $\lambda_{eff}(T)$ >> W we can neglect the presence of magnetic field induced by the superconducting currents, i.e. $\left|\vec{A}\right|=0$. Additionally "bridge" boundary conditions were applied to the ends of the one-dimensional line, i.e.

$$\left|\Psi\left(\pm L/2,t\right)\right|=\sqrt{1-T}$$

$$\Psi\left(\pm L,t+dt\right)=\Psi\left(\pm L/2,t\right)e^{-i\phi\left(\pm L/2,t\right)dt}$$

$$\phi\left(-L/2,t\right)=0$$

and initial conditions were set $\left|\Phi\left(x,0\right)\right|=\sqrt{1-T}$ and $\phi(x,0)=0$.

Simulations were made for parameters, L = 150ξ_0, u = 5.79, T = 0, γ= 40. The value of γ is correlated with the relaxation time of the pairing mechanism. For this iron-based superconductor γ = 40 corresponds to a realistic order of magnitude for this parameter. **Supplementary Figure 8** presents a snapshot the order parameter distribution and the electrical potential along the domain. The order parameter is highly depleted in a fix number of points, and as times passes the value of the order parameter in these points diminishes until it reaches zero. At this time a phase slip center is formed and later the value of the order parameter becomes finite and increases until it reaches a maximum value, marking the end of another periodic cycle. In **Supplementary Figure 9**, we can observe *I-V* curves taken from simulation for a fixed applied current. We note that in these units j_c = 0.38j_0. If we apply a current above critical current the order parameter starts to be spatially modulated and then later phase slips centers nucleate in the middle of the sample (due to symmetry reasons), and afterwards these centers become equally spaced and appear periodically in time. As we decrease current the period of oscillation becomes longer and the number of phase slips becomes lower until it reaches a critical value where all the phase slips disappear. However,

if we start by applying a low current (much below critical current) no signs of phase slips are shown and as we increase current, at some critical value, phase slip centers start to nucleate near the boundary of the sample. As the phase slip centers enter the sample a sudden increase on voltage is observed, as in an avalanche process, in opposition to what is observed in the process going from high to lower currents where the voltage drop with the current decrease is a much smoother process. We also experimented coupling the set of equations to the temperature diffusion equation,

$$C_{eff}\frac{\partial}{\partial t}T = k_{eff}\Delta T + \left|\vec{j}_n\right|^2 - h_c\left(T - T_{(0)}\right),$$

in order to observe the influence of the thermal conductivity. In these equations,

$$C_{eff} = D_s C_s / d_f + C_f$$

$$k_{eff} = \left(D_s k_s / d_f + k_f\right) T_c \sigma_n / \xi^2\left(0\right) j_0^2$$

where h_c is the heat transfer coefficient, T_0 is the applied temperature, C_s, C_f, k_s and k_f are the heat capacity and heat conductivity of the substrate and of the sample, respectively. The initial and boundary conditions applied to this equation were $T(x, 0) = T_0$ and $T(\pm L/2, t) = T_0$. In preliminary results, we observed an increase in the jumps when considering this equation. Furthermore, ref. [10] and Ref. [12] present studies on the behavior of phase slip centers when considering: coupling with the heat diffusion equation, non-uniform values of T_c and of width of the wire, the presence of defected. Fluctuation processes, such as thermal fluctuations, should be also taken into account on later studies, yet it is expected that they help nucleate phase slips centers that are, then, maintained by the applied electrical current. Comparing the theoretical study with the experimental curves, we conclude that the hysteretic and stair like behavior presented in the experimental I - V curves are watermark indications for phase slips centers/lines. However, from the simulation we could not reproduced the big steps in the I - V curves of **Figure 2** of the main text. These big steps in the low to higher current and high to lower current curves are likely related to pinning of phase slip centers that increase the step characteristic of the I - V (with higher steps and larger plateaus) due the avalanche process of entering and leaving of phase slip centers.

Supplementary References

[1]. Li, J. *et al.* Direct observation of the depairing current density in single-crystalline $Ba_{0.5}K_{0.5}Fe_2As_2$ microbridge with nanoscale thickness. *Appl. Phys. Lett.* **103** (6), 062603 (2013).

[2]. Tinkham, M. Introduction to superconductivity: Second Edition. Dover Publications, (2004).

[3]. Li, J. *et al.* Superconductivity suppression of $Ba_{0.5}K_{0.5}Fe_{2-2x}M_{2x}As_2$ single crystals by

substitution of transition metal (*M* = Mn, Ru, Co, Ni, Cu, and Zn). *Phys. Rev. B* **85**, 214509 (2012).

[4]. Silhanek, A. V. *et al*. Influence of artificial pinning on vortex lattice instability in superconducting films. *New J. Phys.***14**, 053006 (2012).

[5]. Tinkham, M. *et al*. Hysteretic I - *V* curves of superconducting nanowires. *Phys. Rev. B* **68**, 134515 (2003).

[6]. Pekker, D. *et al.* Stochastic dynamics of phase-slip trains and superconductive-resistive switching in current-biased nanowires. *Phys. Rev. B* **80**, 214525 (2009).

[7]. Tidecks R., Current-induced nonequilibrium phenomena in quasi-one-dimensional superconductors, Springer, NY, (1990).

[8]. Sivakov, A. G. *et al*., Josephson behavior of phase-slip lines in wide superconducting strips, *Phys. Rev. Lett.* **91**, 267001 (2003).

[9]. Larkin A., Varlamov A., Theory of fluctuations in superconductors, OUP Oxford, London, (2005).

[10]. Elmurodov, A. K. *et al*., Phase-slip phenomena in NbN superconducting nanowires with leads, *Phys. Rev. B* **78**, 214519 (2008).

[11]. Kramer, L., Watts-Tobin R. J., Theory of dissipative current-carrying states in superconducting filaments, *Phys. Rev. Lett.* **40**, 1041 (1978).

[12]. Michotte, S., Condition for the occurrence of phase slip centers in superconducting nanowires under applied current or voltage, *Phys. Rev. B* **69**, 094512 (2004).
